# A Bayesian network based study on determining the relationship between job stress and safety climate factors in occurrence of accidents

**DOI:** 10.1186/s12889-021-12298-z

**Published:** 2021-12-07

**Authors:** Amir Hossein Khoshakhlagh, Saeid Yazdanirad, Masoud Motalebi Kashani, Elham Khatooni, Yaser Hatamnegad, Sohag Kabir

**Affiliations:** 1 Department of Occupational Health Engineering, Faculty of Health, Kashan University of Medical Sciences, Kashan, Iran; 2 School of Health, Shahrekord University of Medical Sciences, Shahrekord, Iran; 3 Social Determinants of Health Research Center, Shahrekord University of Medical Sciences, Shahrekord, Iran; 4 Occupational Health & Safety Department, Kashan University of Medical Sciences, Kashan, Iran; 5 Department of Epidemiology and Biostatistics, School of Public Health, Tehran University of Medical Sciences, Tehran, Iran; 6 Students’ Scientific Research Center, Tehran University of Medical Sciences, Tehran, Iran; 7 Department of computer science, University of Bradford, Bradford, BD7 1DP UK

**Keywords:** Job stress, Safety climate, Accident, Bayesian network

## Abstract

**Background:**

Job stress and safety climate have been recognized as two crucial factors that can increase the risk of occupational accidents. This study was performed to determine the relationship between job stress and safety climate factors in the occurrence of accidents using the Bayesian network model.

**Methods:**

This cross-sectional study was performed on 1530 male workers of Asaluyeh petrochemical company in Iran. The participants were asked to complete the questionnaires, including demographical information and accident history questionnaire, NIOSH generic job stress questionnaire, and Nordic safety climate questionnaire. Also, work experience and the accident history data were inquired from the petrochemical health unit. Finally, the relationships between the variables were investigated using the Bayesian network model.

**Results:**

A high job stress condition could decrease the high safety climate from 53 to 37% and increase the accident occurrence from 72 to 94%. Moreover, a low safety climate condition could increase the accident occurrence from 72 to 93%. Also, the concurrent high job stress and low safety climate could raise the accident occurrence from 72 to 93%. Among the associations between the job stress factor and safety climate dimensions, the job stress and worker’s safety priority and risk non-acceptance (0.19) had the highest mean influence value.

**Conclusion:**

The adverse effect of high job stress conditions on accident occurrence is twofold. It can directly increase the accident occurrence probability and in another way, it can indirectly increase the accident occurrence probability by causing the safety climate to go to a lower level.

## Background

The International Labor organization describes workplace accidents as unplanned and unpredictable events that create obvious injuries [1]. Based on the statistics, over 264 million occupational accidents and 350,000 mortalities due to those occur every year in the world [2]. The financial burden of occupational accidents and diseases in compared to that of costly Diseases such as cancers, Alzheimer, human immunodeficiency virus (HIV), and cardiovascular illnesses is higher [3]. Different studies have been investigated the causes of accidents. Some of these agents included individual, organizational, environmental, social, job factors [4]. Of effective factors, job stress and safety climate play important roles in the occurrence of accidents.

Job stress, as one of the most common occupational problems, seriously threatens staff health in the world. The results of studies show that higher levels of job stress can directly affect the body’s immune system and mental health. This stress also causes disorders and illnesses such as depression and anxiety, immune deficiency disorders, headaches, musculoskeletal pain, and cardiovascular disease [5, 6]. Furthermore, it can affect individual and organizational performances [7]. Based on the results of studies, occupational stress affects productivity, absenteeism, burnout, and job turnover [8, 9]. There are various definitions of job stress in the literature. McGrath and Altman state that stress is the fundamental imbalance between an individual’s capability and job demand that leads to severe consequences [10]. There are different occupational factors affecting job stress, such as job control, conflict at work, job satisfaction, mental demand, physical environment, social support, and workload and responsibility [11]. If there are no coordination among job requirements, abilities, and desires, then stress can lead to many behavioral, physical, and psychological consequences. The International Labor Organization estimates the costs of job stress to be 1 to 3.5% of the Gross Domestic Product (GDP) of each country [12]. The study results in the developed countries indicate that nearly 30% of the staff suffer from job stress [13]. In addition to the mental and functional consequences, job stress can lead to unsafe behaviors and accidents through impaired focus and decision-making. The results of several studies have proven that stress plays a vital role in 37% of industrial accidents & injuries [13].

Safety climate is another critical factor affecting staff health and accident occurrence. Safety climate defines the workers’ perceptions of the organizational prioritization related to workplace safety compared to business demands [14–16]. Accordingly, safety climate has various attributes such as management’s commitment to safety and safety communication, encouraging the workers to the safety behavior in order to promote safety and performance outcomes [16, 17]. The results of several meta-analysis studies show that the safety climate affects safety outcomes such as accidents and injuries, as well as safety compliance and participation behaviors [18, 19]. The results of a review study performed by Kalteh et al. also showed that increasing the level of safety climate can reduce accident incidence rate and improve safety performance [20]. The planned behavior theory describes the role of the safety climate in causing safety behavior [21]. Based on this theory, three central components, including attitude toward the behavior, subjective norms, and perceived behavioral control, are effective in creating unsafe behavior. Fogarty and Shaw demonstrated that the management attitude and safety climate significantly affect these three components and regulate the behavior [21]. Moreover, the safety climate can make the accident occurrence through other paths such as job stress, which can affect job satisfaction, staff engagement, and turnover [22]. Dollard et al. stated that a psychosocial safety climate adjusts the job demand – resource interaction and controls job stress [23]. However, there may also be a reverse path from work stress toward a safe climate. In other words, job stress factors may affect the safety climate and lead to accidents through this path. This assumption can be examined using the Bayesian network model. This type of model has been exploited to analyze the health and safety issues in previous studies. Mohammadfam et al. applied the Bayesian network model for modeling and evaluating the safety behavior of the workers in the industry [24]. Mirzaei et al. represented a novel procedure for risk modeling of a hydrogen gasholder using the Fuzzy Bayesian Network [25]. Herrero et al. used the Bayesian network models for analyzing the effect of working conditions and psychological/physical symptoms on occupational accidents [26]. Ahn et al. exploited the Bayesian network model for diagnosing the relations between work-related musculoskeletal disorders and working characteristics [27]. In the present study, it was aimed to determine the relationship between job stress and safety climate dimensions in the occurrence of accidents using the Bayesian network model.

## Material and methods

**Fig. 1:**
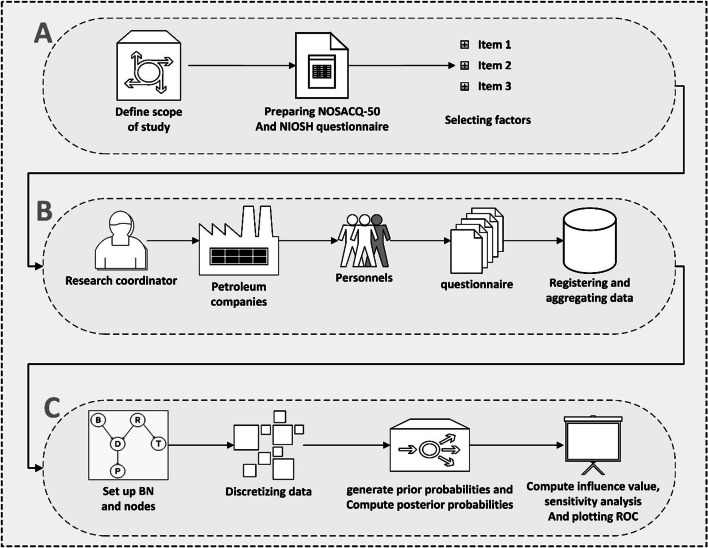
The workflow of the method used in this paper

Figure 1 shows the workflow of the method used in this paper. The total steps of this study included preparing questionnaires, collecting data, and data analysis. The authors confirm that all methods were carried out in accordance with relevant guidelines and regulations of Helsinki.

## Participants

This cross-sectional study was performed on 1530 male workers of Asaluyeh petrochemical company in Iran, from August 2018 to May 2019. The study population consists of the staff employed in various departments, including technical, electrical, machinery, maintenance, mechanical, welding, turning, and supervision units of the petrochemical company. Firstly, a list of people working in each department was created, and subjects were randomly selected from the list. All participants declared their informed consent, using the consent form provided by the medical ethics committee of Tehran University of medical sciences. Before the beginning of the study, the test stages were clearly explained to the participants. Inclusion criteria were having work experience for more than one year and being literate. Exclusion criteria were lack of cooperation and lack of focus on completing the questionnaires.

## Data acquisition

Before the beginning of the study, the goals and stages of the study were clearly explained to the participants. Then, they were given the necessary training on how to complete the questionnaires. The questionnaires were completed in the presence of the researchers at the rest times. The questionnaires included demographical information, accident history questionnaire, NIOSH Generic Job Stress Questionnaire (GJSQ), and Nordic Safety Climate Questionnaire (NOSACQ-50). Also, work experience and the accident history data were inquired from the petrochemical health unit.

## Data collection instruments

### Demographic data and accident history questionnaire

The demographic data including age, education, marital status, job type, working department, working experience, body mass index, and smoking were collected using a researcher-made questionnaire. Also, participants were questioned about their accident experience and accident type at workplaces during the past year.

### NIOSH generic job stress questionnaire (GJSQ)

NIOSH Generic Job Stress Questionnaire (GJSQ) developed by the US National Institute for Occupational Safety and Health (NIOSH) was used to measure the different job stress factors among workers. Kazronian et al. [11] have evaluated and validated this questionnaire in Iranian firefighters. They claimed that the Cronbach Alpha Coefficient of this questionnaire is more than 0.7, and its Intra-Cluster Correlation Coefficient is 0.7. However, in the present study, the Cronbach Alpha Coefficient of the questionnaire and the Intra-Class Correlation Coefficient were calculated again. NIOSH Generic Job Stress Questionnaire (GJSQ) encompasses 21 parts or factors; including background information (7 questions), conflict at work (16 questions), employment opportunities (4 questions), general health (17 questions), health conditions (24 questions), feelings about his-self (10 questions), general job information (12 questions), job requirements (10 questions), job satisfaction (4 questions), mental demands (5 questions), non-work activities (7 questions), other health information (33 questions), physical environment (10 questions), problems at work (6 questions), social support (12 questions), work hazards (5 questions), work limitations (5 questions), workload and responsibility (11 questions), job (14 questions), and job future (5 questions). The answer options are different, including yes or no, false and true, Likert from one to five, Likert from one to three, and open-ended answers [28]. The total score was calculated with the mean value of the scores in job stress scales.

### Nordic safety climate questionnaire (NOSACQ-50)

It is a validated questionnaire on safety climate survey. A team of occupational safety experts from several Nordic countries including Denmark, Norway, Iceland, Finland, and Sweden led by Pete Kines from the National Research Centre for the Working Environment (NRCWE) developed the questionnaire in 2011 [29]. This questionnaire has 50 questions and 7 subscales; including management’s safety priority and ability (9 questions), management’s safety empowerment (7 questions), management’s safety justice (6 questions), worker’s safety commitment (6 questions), worker’s safety priority and non-acceptance risk (7 questions), peer safety communication, learning, and trust (8 questions), and worker’s trust in the efficacy of safety systems (7 questions) [29]. Yousefi et al. [30] in 2016, have evaluated the validity and reliability of this questionnaire in Iran. Alpha Cronbach Coefficient of this questionnaire obtained was 0.94. However, in the present study, Cronbach Alpha Coefficient and the Intra-Class Correlation Coefficient were calculated again. In this questionnaire, the questions are answered via Likert ranging from one to four; including strongly disagree, disagree, agree, and strongly agree. The total score was calculated with the mean value of the scores in job stress scales.

## Data analysis

### Statistical tests

*First, we discretized the any of understudy variables (*the safety climate dimensions and occupational stress scales*) with the Uniform-Width method* into a dichotomous variable (low and high)*.* Then, the statistical tests were carried out using SPSS software version 24. Descriptive statistics were calculated. Then the expectation-maximization method was applied to calculate and replace the missing values. Cronbach Alpha Coefficient and Internal Correlation Coefficient calculated by a two-way mixed analysis of variance. The result of this analysis was used for evaluating the validity and reliability of the questionnaires. Besides, the bivariate and multivariate correlations coefficients for the job stress and safety climate variables were calculated.

### Bayesian network modeling

Bayesian Networks (BNs) are probabilistic and graphical models introduced by Pearl. In the present study, GeNIe academic software version 2.3 (university of Pittsburg) with an expert-knowledge base method was used. Five experts were asked to give their opinion on three possible relationships (A↑B, A → B, and A ← B) and for this purpose, the Dempster-Shafer theory was applied [31]. After creating the BN graphical structure, a Conditional Probability Table (CPT) was obtained from the developed model with the Expectation-Maximization algorithm [32]. In the next step, delta p sensitivity analysis was used to rank the factors corresponding to accident state [24], and finally,10-fold cross-validation analysis was also used to evaluate the model. Dataset was randomly divided into ten equal folds, and in any 10-repeat cross-validation, nine folds (9 subsamples) were used to train the Bayesian network model, and the remaining fold (1 subsample) was used to validate the model. A sensitivity analysis also was performed to investigate the effects of the statistically significant variables [33].

## Results

According to the analysis of the collected data, the internal consistency of all dimensions is at an excellent level (Cronbach’s alpha> 0.9). Also, based on the results of the two-way mixed model, the intra-class correlation coefficients for the reliability of a single rating, and the aggregated mean have excellent values. Table 1 shows the descriptive information of the participants’ demographic variables. Most of the subjects were 30 to 39 years old (50.7%), at high school education level (44.9%), 5 to 10 years of job experience (47.3%), with body mass index higher than 25 (54.8%), repairing and machinery job type (36.2%), married (84.4%), and smoker (58.2%). Table 2 also represents the Conditional Probability Table (CPT) for accident state, which reports the relationship coefficient among the variables.

**Table 1:** The descriptive information of the participants’ demographic variable

Demographic variable		Frequency	Valid Percent
Age (years)	20 - 29	226	14.8
	30 -39	775	50.7
	40 - 49	471	30.8
	50 - 59	58	3.8
Education degree	Under diploma	247	16.1
	diploma	687	44.9
	Associate degree	460	30.1
	Bachelor Degree	127	8.3
	Master’s degree	9	0.6
Job experience (years)	1-5	124	8.1
	5-10	724	47.3
	10-15	427	27.9
	15 and higher	255	16.7
Body mass index	17.5 – 20	105	6.9
	20 – 25	585	38.3
	25 and higher	837	54.8
Type of job	Technical worker	150	9.8
	Electrical worker	131	8.6
	Machinery worker	273	17.8
	Repairing worker	281	18.4
	Conversion worker	261	17.1
	Turnery worker	187	12.2
	Welding worker	128	8.4
	Mechanic worker	64	4.2
	supervisor	55	3.6
Marital status	single	238	15.6
	married	1292	84.4
Smoking	yes	891	58.2
	no	639	41.38

**Table 2:** CPT for “accident state”

Safety climate	Job stress	yes	no
low	low	0.91	0.09
low	high	0.93	0.07
high	low	0.32	0.68
high	high	0.95	0.05

**Fig. 2:**
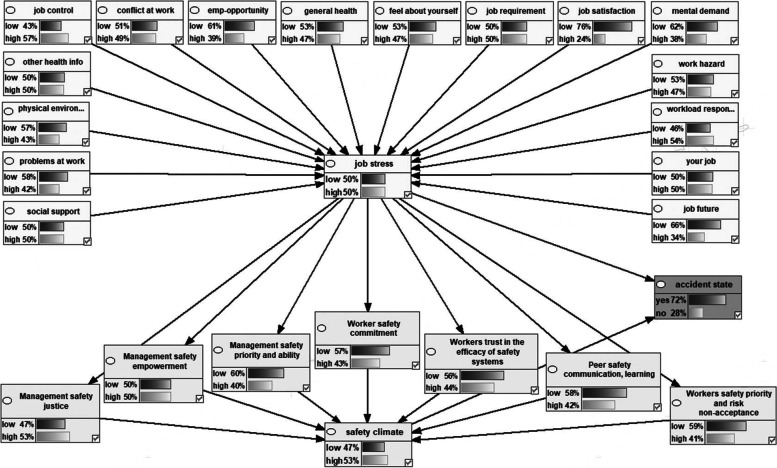
Bayesian network model representing the dependencies among the variables the marginal probabilities of the studied variables

**Fig. 3:**
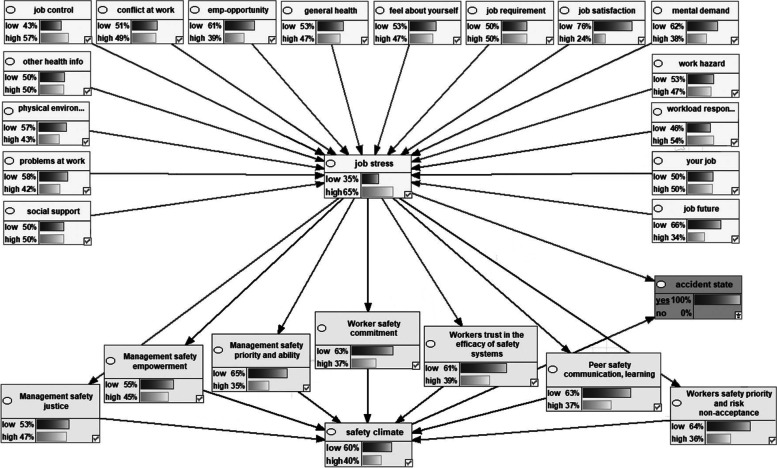
Sensitivity analysis of accident occurrence

**Fig. 4:**
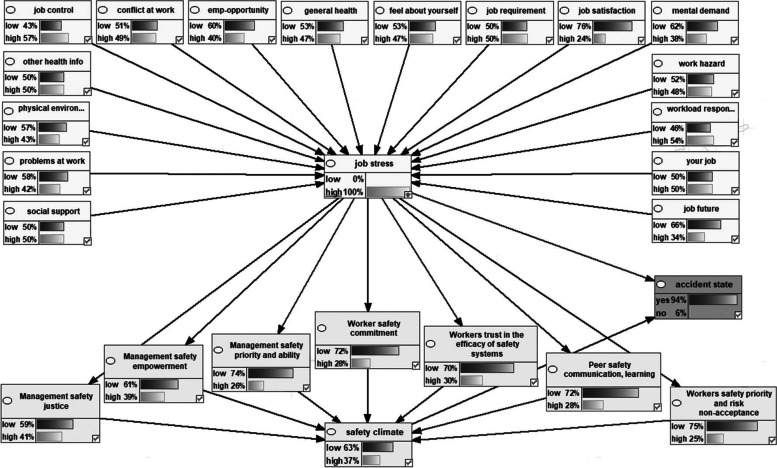
Sensitivity analysis of job stress

**Fig. 5:**
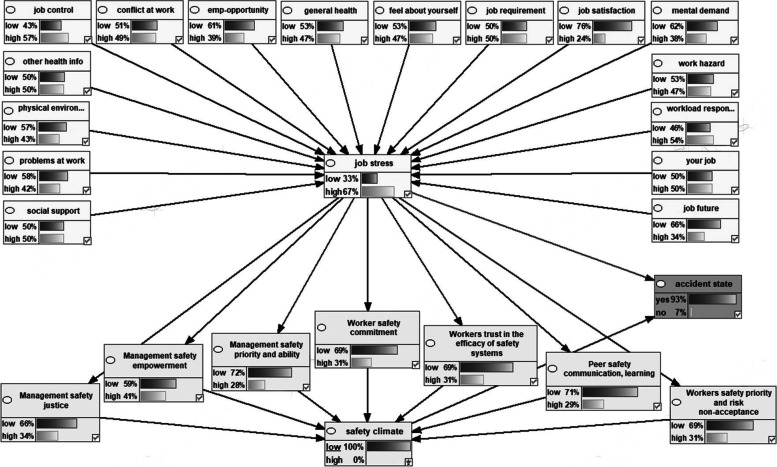
Sensitivity analysis on safety climate

**Fig. 6:**
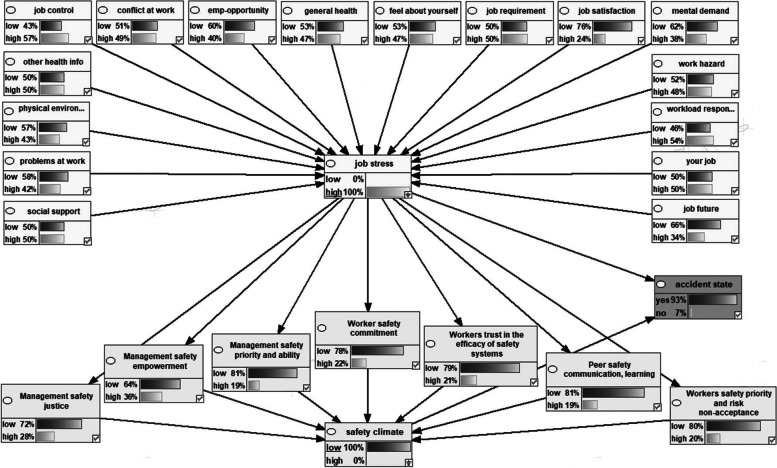
Sensitivity analysis on simultaneously safety climate and job stress

Figure 2 shows the Bayesian network model representing the dependencies among the variables the marginal probabilities of the studied variables. Figures 3, 4, 5, 6 also indicate the sensitivity analyses. At the occurrence of the accident with the probability of 100%, the probability of the high job stress enhanced from 50 to 65%, and the probability of the high safety climate decreased from 53 to 40%. For safety climate dimensions, most decreases were related to the management’s safety justice from 53 to 47% and the worker’s safety commitment from 43 to 37%. For the high job stress condition with the probability of 100%, the probability of the high safety climate decreased from 53 to 37% and the probability of the accident occurrence increased from 72 to 94%. For safety climate dimensions, most decreases were related to the worker’s safety priority, and risk non-acceptance from 41 to 25% and the worker’s safety commitment from 43 to 28%. For the low safety climate condition with the probability of 100%, the probability of the accident occurrence increased from 72 to 93%. For the safety climate dimensions, most decreases were related to the management’s safety justice from 53 to 34%. Besides, at the concurrently low safety climate and high job stress with the probability of 100%, the probability of accident occurrence increased from 72 to 93%. Lastly, for the safety climate dimensions, most decreases were also related to the management’s safety justice from 53 to 28%.

Table 3 demonstrated ranked posterior probabilities of occurring accidents. As Table 4 shows, the calculated values are from the associations between factors. The most effective mean values were related to the job stress and accident occurrence association (0.39), the safety climate and accident occurrence relation (0.34), and the management’s safety justice and safety climate (0.19). For the association between the job stress factor and safety climate dimensions, the job stress and worker’s safety priority and risk non-acceptance relation (0.19) had the highest mean value. For the job stress factor and job stress scales associations, the associations of the job stress factor with general health (0.004), employment opportunity (0.004), and other health information (0.003) showed the highest mean values.

**Table 3:** Predicted probability of “occupational accident = no” (%) corresponded to other factors states

Safety Climate and Job Stress Dimensions	Low %	High %	Variation^†^	Rank
Job stress	78.01	− 77.72	155.73	1
Safety climate	−73.05	66.37	139.42	2
Peer safety communication, learning, and trust	−26.04	35.37	61.41	3
Management’s safety justice	−32.19	28.27	60.45	4
Management’s safety priority and ability	−23.11	34.13	57.25	5
Worker’s safety commitment	−24.13	32.60	56.73	6
Worker’s trust in the efficacy of safety systems	−24.30	30.68	54.98	7
Worker’s safety priority and non-acceptance risk	−22.06	32.14	54.20	8
Management’s safety empowerment	−23.49	23.51	47.00	9
General health	0.33	−0.37	0.70	10
Work hazard	0.30	−0.33	0.63	11
Other health info	0.30	−0.30	0.60	12
Employment opportunity	0.22	−0.34	0.56	13
Your job	0.24	−0.25	0.49	14
Mental demand	0.18	−0.30	0.48	15
Workload responsibility	0.24	−0.21	0.44	16
Job requirement	0.22	−0.22	0.43	17
Problems at work	0.14	−0.19	0.33	18
Job control	0.16	−0.12	0.27	19
Feel about yourself	0.10	−0.12	0.22	20
Job satisfaction	−0.04	0.13	0.17	21
Social support	0.08	−0.08	0.16	22
Conflict at work	0.08	−0.08	0.16	23
Job future	−0.04	0.08	0.13	24
Physical environment	−0.01	0.01	0.02	25

^†^ *Variation* = |*Low*%| + |*High*%|

**Table 4:** The calculated influence values from the association of the factors in developed model

Parent	Child	Influence value
Job stress	Accident state	0.39
Safety climate	Accident state	0.34
Management’s safety justice	Safety climate	0.19
Job stress	Worker’s safety priority and risk non-acceptance	0.19
Job stress	Management’s safety priority and ability	0.18
Job stress	Peer safety communication, learning, and trust	0.17
Job stress	Worker’s safety commitment	0.17
Job stress	Worker’s trust in the efficacy of safety systems	0.16
Peer safety communication, learning, and trust	Safety climate	0.15
Management’s safety priority and ability	Safety climate	0.14
Worker’s trust in the efficacy of safety systems	Safety climate	0.14
Job stress	Management’s safety justice	0.13
Worker’s safety commitment	Safety climate	0.13
Job stress	Management’s safety empowerment	0.11
Worker’s safety priority and risk non-acceptance	Safety climate	0.11
Management’s safety empowerment	Safety climate	0.09
General health	Job stress	0.004
Employment opportunity	Job stress	0.004
Work hazard	Job stress	0.003
Other health information	Job stress	0.003
Mental demand	Job stress	0.003
Your job	Job stress	0.002
Workload responsibility	Job stress	0.002
Job requirement	Job stress	0.002
Problems at work	Job stress	0.002
Job control	Job stress	0.002
Job satisfaction	Job stress	0.001
Feel about yourself	Job stress	0.001
Conflict at work	Job stress	0.001
Social support	Job stress	0.001
Job future	Job stress	0.001
Physical environment	Job stress	0.0001

**Fig. 7:**
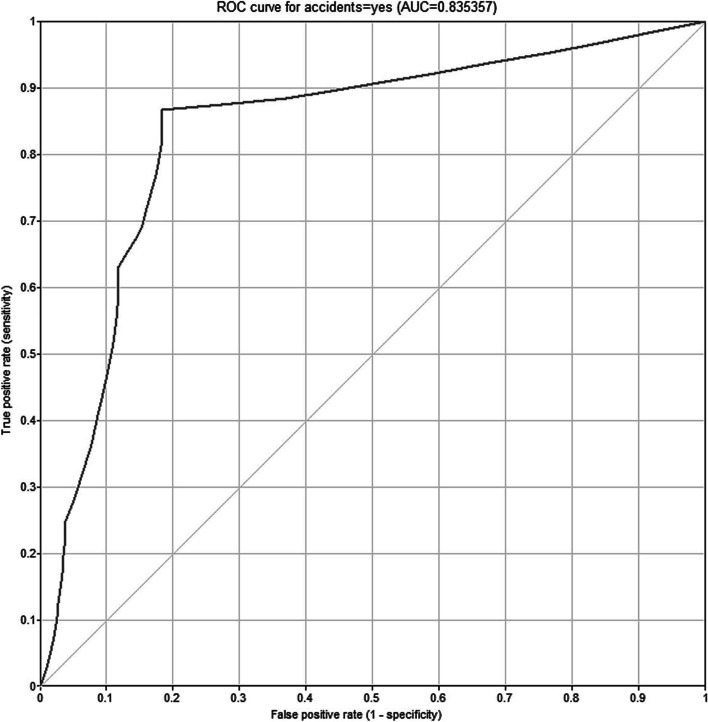
The ROC curve

A ROC curve was drawn to investigate the validity of the fitted Bayesian model, and the area under the curve was calculated (Fig. 7). This value was 0.8353. The parameters of the confusion matrix related to the classification of the occupational accident status also were computed (Table 5). The values of the sensitivity, specificity, and accuracy of the model were equal to 87, 82, and 85%, respectively.

**Table 5:** The confusion matrix related to the classification of the occupational accident status

		predicted		
		yes	no	
actual	yes	TP = 988	FN = 151	Sensitivity = 87%
	no	FP = 72	TN = 319	Specificity = 82%
				Accuracy = 85%

## Discussion

In the present study, a Bayesian model was fitted to analyze the inter-association between job stress, safety climate, and accident occurrence. This model has been used to clarify how job stress can affect accident occurrence, directly and indirectly, through the safety climate. The results showed that the presented model has good validity. The validity and reliability of the used questionnaires were investigated and were accepted as valid and reliable tools. In general, the results obtained from the model showed that a high job stress condition could decrease the high safety climate from 53 to 37% and increase the accident occurrence from 72 to 94%. Analysis of the other paths in the model also demonstrated that a low safety climate condition could increase the accident occurrence from 72 to 93%. Also, the concurrent high job stress and low safety climate could raise the accident occurrence from 72 to 93%. These results show that two factors of job stress and safety climate play an essential role in the accident occurrence.

Job stress has a significant impact on the occurrence of accidents both directly and indirectly through decreasing the safety climate. Several studies have indicated this path as a direct impact. Barkhordari et al. represented a model that occupational stress can lead to individual failure and carelessness, which can increase unsafe acts or conditions and thereby cause accidents and injuries [34]. The high job stress and imbalance between individual capability and job demand reduce the person’s cognitive performance and create cognitive failure. Therefore, individuals cannot decide and act correctly [35]. Chou et al. concluded that perceived occupational stress decreases the cortical activity of the prefrontal cortex [36]. According to Mohammadfam et al., there is a significant direct association between job stress and unsafe acts and accidents. Therefore, the increase of one score in occupational stress enhances the unsafe acts by 1 % [37]. Julia et al. also investigated the relationship between occupational stress and accident occurrence and concluded that there is a significant positive association between them [38]. The results of the present study are compatible with the stated studies. The indirect effect path of the job stress on the accident occurrence was through the effect on the safety climate.

Occupational stress appears to affect the safety climate through two probable significant paths. Job stress can cause physical and psychological consequences. Chronic stress and adverse working conditions cab be resulted in job burnout and cause physical and psychological consequences. The physical effects include hypercholesterolemia, type 2 diabetes, coronary heart disease, hospitalization due to cardiovascular disorder, musculoskeletal pain, changes in pain experiences, prolonged fatigue, headaches, gastrointestinal issues, and respiratory problems. The psychological effects consisted of insomnia, depressive symptoms, use of psychotropic and antidepressant medications, hospitalization for mental disorders, and psychological ill-health symptoms. Job dissatisfaction, absenteeism, new disability pension, job demands, job resources, and presenteeism [39]. The results of a study performed by McCaughey et al. showed that workplace injuries and illnesses had negative effects on perceptions of safety climate [40]. On the other hand, job stress may provoke negative feelings in the workplace [41]. These feelings can impress on the job satisfaction of the workers. Stoilkovska et al. concluded that job satisfaction had a significant effect on the perceived safety climate [42].

The results of the present study showed that at the concurrent high job stress and low safety climate situation, the management’s safety justice has the most decrease from 53 to 28% among the safety climate dimensions. On the other hand, the perception of the health problem creates job stress and affects the safety climate. In the current study, however, the relationships between the job stress factor and job stress scales, the relations of the job stress factor with general health, work hazard, and other health information showed the highest mean influence values. McCaughey et al. demonstrated that workplace injury and illness have adverse effects on the safety climate perceptions and health care worker outcomes [40]. Hall et al. also concluded that the safety climate modulates the impact of the job demand on depression and further the effect of the depression on the positive organizational behaviors [43].

Also, the results of the present study indicated that at the high job stress condition, most decreases were related to the worker’s safety priority and risk non-acceptance, and the worker’s safety commitment. Among the associations between the job stress factor and safety climate dimensions, the job stress and worker’s safety priority and risk non-acceptance had the highest mean influence value. The results of Griffin et al. study revealed that job stress has a significant positive association with depersonalization and a negative relationship with organizational commitment [44]. Bronkhorst also stated that the job demands, job resources, and safety climate affect the physical and psychological safety behavior and the safety climate has the mediator role in the association between job demands and job resources with the safety behaviors [45]. These results are compatible with the results of the present study.

Limitations of the present study included the lack of data analysis in different parts of the industry and job positions. Also, the effect of non-occupational stress due to family and community environments was not investigated, and the effect of demographic variables on the presented model was not studied. Therefore, it recommends that these limitations be solved in future studies. Besides, the effect of the proposed model on the number and severity of unsafe behaviors and the number and severity of work accidents were not considered, which authors will investigate it in subsequent studies. Lastly, this study applies the self-report instruments as the data source, which may cause some bias in the results.

## Conclusions

In this paper, we utilize the flexible modeling and inference capability BN to perform a study to investigate the effects of job stress and safety climate on the occurrence of an accident. The results of this study showed that the adverse effect of high job stress conditions on accident occurrence is twofold. It can directly increase the accident occurrence probability and in another way, it can indirectly increase the accident occurrence probability by causing the safety climate to go to a lower level. Therefore, to improve the perceived safety climate and decrease the accident occurrence probability, the organizations should put more effort to reduce the job stress on the employee. Some of the strategies for handling the stress in the workplaces included providing a balance between demands and resources, increasing social supports, increasing job control, decreasing role pressures, increasing job satisfaction, increasing job security, and strengthening problem-oriented coping style.

## Data Availability

All data generated and analyzed during this step of the study are included in this published article.

## Declarations

### Declaration of Helsinki

The authors confirm that all methods were carried out in accordance with relevant guidelines and regulations.

## Abbreviations

GDP: Gross Domestic Product
GJSQ: Generic Job Stress Questionnaire
NOSACQ-50: Nordic Safety Climate Questionnaire
NRCWE: National Research Centre for the Working Environment
BN: Bayesian Networks
CPT: Conditional Probability Table
